# Twenty Paces from History: An Interview with Soraya de Chadarevian

**DOI:** 10.1371/journal.pgen.0020162

**Published:** 2006-09-29

**Authors:** Jane Gitschier

What is it about Cambridge that so delights me? Could it be the soothing rhythm of punts and poles upon the Cam? The fan vaulting of King's chapel, rendered even more breathtaking by the dappling of light through stained glass? The rickety Dutch bikes spilling into narrow streets as residents sensibly eschew motor power? Or its lure as birthplace of molecular biology, a modern chapter in the lineage of scientific inquiry nucleated here by the likes of Newton and Darwin? The answer is all of the above.

Each time I visit Cambridge, I succumb to the magnetism of Free School Lane, and like an iron filing I'm pulled deeper and deeper into the interior courtyard of the Cavendish labs. It was here, on the “first floor” of the Austin wing, where Watson joined Crick and the structure of DNA was deduced. Last year, I prevailed upon an affable ginger-haired woman, who worked in a hut nearby, to direct me to this hallowed space. She seemed to take delight in my quest and pointed out a window in the large yellow-brick structure. “There,” she said, her friend worked in the very room where the model was built. I looked up and knew she was right.

To learn more, I turned to the Department of History and Philosophy of Science, adjacent to the Cavendish, and was referred to the book *Designs for Life,* a history of moleculary biology in Cambridge, written by one of their faculty, Soraya de Chadarevian. I darted over to the Cambridge University Press bookshop, picked up a copy, and dove into it on my way back to the United States. One year later I took a sabbatical at the Wellcome Trust Sanger Institute, grandchild of the noble Cavendish, and I had the opportunity to visit the author in her small office overlooking history. What, I wondered, was it like to immerse oneself in that remarkable period?

**Jane Gitschier:** How did you happen to come to Cambridge?

**Soraya de Chadarevian:** I came here fifteen years ago on a three-year Wellcome fellowship to work on a project on the history of molecular biology in Cambridge.

The Wellcome Trust funded several Units for the History of Medicine, and one Unit was here in Cambridge, under one roof with the Department of History and Philosophy of Science. The Unit had decided to launch this topic as a project. They were looking for a researcher to do the main work on the project. They hired me.

**JG:** What was your background?

**SDC:** My first degree was in biology and then I did my PhD in philosophy. I had just done a post-doc in history of science in Berlin. I was working on a 19th century project, and I liked that a lot. But it was suggested to me that if I wanted to stay in the history of science, I should consider moving to the 20th century so that I could use my science skills.

This is always an issue: do you need to have a science background when you work in the history of science, or don't you? I think either is possible, but for me it has been a big advantage that I had science background.

First, it was easier that I didn't have to learn the science, but also I personally think it is difficult to write the kind of things I write without having been in the lab, without having an idea of what it means to do science. And when you interview scientists, they talk to you in a very different way if they know that you understand what they say. I think they respect you more. I think it has been crucial that I have a science degree.

**JG:** Where did you study?

**SDC:** I did a Diplom in Biology in Germany. I grew up in Italy, but I did most of my studies in Germany.

**JG:** But your name is not Italian.

**SDC:** My first name is Arabic, the last name is Armenian. But my mother was German, so I went to the German School in Rome and then won a fellowship to study at the University of Freiburg. The Diplom is a five-year course, which includes an extra year of experimental work, which I did in Bologna.

**JG:** What kind of research did you do?

**SDC:** I worked on bioenergetics, on the mechanism of ATP formation in the cell. A group at the University of Warwick in the United Kingdom was promoting a chemical-coupling hypothesis, in contrast to the electrochemical hypothesis proposed by Mitchell. In the chemical-coupling hypothesis, there was one essential ingredient—lipoic acid—so we worked on bacterial mutants that were lipoic-acid deficient.

It was fantastic being a graduate student, having a project that was your own. And it was really a clear-cut question, a textbook question. And, actually, my experience of how science works was very important for my future work on the history of science. For instance, we got our results very quickly and clearly, but we didn't believe them, because they contradicted the results of this other group in Warwick.

A simple experiment became excessively complicated, because the group from the other lab suggested that lipoic acid could linger in trace amounts and that we were not working cleanly enough. Or, perhaps, that the water in Bologna was different from the water in Warwick. A research assistant came down to Bologna to work with me, because the idea was that we were doing something different, something that couldn't really be figured out from the recipes: the idea of tacit knowledge that goes into doing an experiment but not written into the methods section of papers. It is very relevant for the approaches in history of science, which I was to learn later, but here I was living it!

Still, we couldn't reproduce the results of the competing group. In the end, it turned out that theirs was a case of fraud! Then there was another group, Canadian, that got results similar to ours. At that point we very quickly published our results.

For me, this was a lesson in how science works and how knowledge is made. Sociologists have called it the “experimenter's regress.” It means that when you do an experiment and get some unexpected results, you don't know whether to trust the results or to suspect that something is wrong with the way the experiment was done. And you can't decide in advance.

During that time, I became interested in thinking about how science works, how knowledge is produced, and about the place of science in society more generally. At that time this meant going into philosophy, because history of science in Germany at the time was much more of the antiquarian sort—who invented what and when—and I thought that was boring.

After receiving my PhD, I learned about a German postdoctoral fellowship program aimed at introducing new approaches to the history of science, and I became one of the first generation of fellows in that program in Berlin.

**JG:** So what was this new way of thinking about science?

**SDC:** It came from many directions. The general idea was to look at science as a practice, as a culture. It involved anthropologists moving to the laboratories and looking at scientists as a foreign tribe. They would ask: What are scientists after? How do they talk to each other? And what do they actually do?

It also came from the sociology of science, and from taking seriously the material culture of science, not looking at science just as a system of theories, but as a system of practices. And making links to other cultural practices.

During the 1980s, the history of science became really interesting, because there were people coming from many different disciplines and backgrounds and working together in new ways, examining what scientists did in the lab and beyond. There was a very special dynamic that became extremely productive.

We are talking about the history of this discipline. These new approaches came out of a particular historical moment, when new questions were raised about the role of science in society and about the role of the expert. Many of the people who started to question this were scientists themselves. This had started in the late 1960s with the critique of the role of science and technology in the Vietnam War.

**JG:** Let's now talk about your book.

**SDC:** The project changed dramatically in the course of the time I worked on it. I worked on it for a long time! The gestation period for the research and for writing the book and getting it out was nearly a decade, which many people think is what you need for a historical project. Other things came out, too, including two edited volumes, an exhibition, and a series of articles, all of which were part of the big project.

**JG:** How did the project evolve?

**SDC:** At the beginning it wasn't very well defined at all. The organizers had the feeling that Watson and Crick had been here, so somehow molecular biology started here, and they wanted to see how it established itself as a discipline.

What I did was to place these developments into the broader historical context of where science and scientists were standing at the time. People might be surprised that the first chapter in the book talks about the mobilization of scientists in the war. [Indeed, the subtitle is *Molecular Biology after World War II*]. But I can tell you how I got to that.

There was a small steering board for this project. One of the people on the board was John Kendrew, and he was one of the first people I interviewed. It was in talking to him, and later to others, that the experience of being a scientist in the war was so dominant and so important in their telling of their lives as scientists, that it just struck me that this had to be part of the story I was going to tell. And it struck me particularly in coming from Germany. German scientists would have done everything to avoid that subject! The experiences and skills scientists gained working on war-related projects became important for their careers and for their research after the war.

Also pivotal was that funding for science changed dramatically after the war, as a result of the war. Funding for science projects escalated during the war, and scientists made the case that this funding had to be kept up after the war. They convinced the politicians. In Britain, this became a very strong topic. The scientists had contributed to winning the war—see radar, see the atomic bomb—and they would help the country win the battle of peace, in the rhetoric of the time. Molecular biology didn't exist at the time, but “biophysics” attracted much funding. It included the use of physical technologies, often developed in weapon-related research, for biological and medical purposes.

Today there is a lot of research done on the science of the 1940s and 1950s. There is always a lag time because historians need to wait for the opening of archives, and, in this particular case, they also needed to wait for the end of the Cold War. People are now working, for example, on the history of radioisotopes and how their use in biology and medicine was actively promoted by the Atomic Energy Commission in the US—the peaceful side, the good side, of physics, something that could somehow redeem the destructiveness of the bomb. People were speaking in these terms, suggesting that the medical uses of radiation could save more lives than were lost in Hiroshima and Nagasaki.

Postwar, science funds were expanding and there were opportunities for new research. People were coming back to research after long interruptions in their scientific careers. And they were prepared to move into completely new fields. Instead of choosing a topic that was more or less suggested by their supervisor, they decided to do something they were really interested in, since they had to start from scratch anyway. For example, Francis Crick in his autobiography speaks in such terms. After the war, he wasn't keen on continuing his PhD topic, which he considered “extremely dull,” in his own words. Luckily, he said, the war had destroyed his experimental setup, and so after the war he couldn't go back to it.

When I started my project, I had made one decision: people had written so much about Watson and Crick already, even before the 2003 50th anniversary, that I didn't want to focus on that. I wanted to look further afield.

But eventually I realized that the double helix discovery so dominated the history of the field that I just had to engage with it. Writing the history of a field also means reflecting on the way that history has been written before and on how history is used, for instance, by scientists to construct a particular picture of their science.

In the case of Watson and Crick, the question is why has that story become so important? You have to understand its place and meaning before you can see what other stories you might be able to tell.

**JG:** Well, Watson himself was such a great spokesman for the story.

**SDC:** Of course. But one needs to understand what kind of a story it is. It is not history. Actually, Watson himself called his book *A Personal Account of the Discovery of the Structure of DNA.* He didn't even claim it was history.

**JG:** How do you define history, then?

**SDC:** History is done by looking at events with some distance, by studying the papers and putting things in context. The same is true of general history.

**JG:** But how do you know that papers, and in fact this is where my argument lies for oral history, actually reflect what really happened at the time?

**SDC:** I am talking about papers in a broad sense. The papers kept in archives, including personal archives, institutional papers, and correspondence of the time. Of course papers always need to be placed and understood in the context in which they were produced. They don't tell the story “as it was.”

**JG:** What are we going to do in the future with everything these days being E-mail?

**SDC:** That is a big problem. Archivists are trying to think about that. For instance, John Sulston has kept all of his E-mails and they form part of his archive that will be deposited with the Wellcome Library.

**JG:** Yes, I read Sulston's book *[The Common Thread],* and I was amazed that he cited E-mails from five and ten years ago.

**SDC:** Sulston used E-mail systems early on. They were crucial for communicating in the collaborative networks he was working in, and he was aware of the importance of that. There is an extremely interesting history to be written about this early electronic communication between scientists and how that affected the kind of science that could be done. So that is the kind of question we are interested in.

The human genome project poses other questions as well. For instance, where is the discovery in that kind of research? This has been the problem in high-energy physics all along, where hundreds of people are involved in single experiments. Who is the author? Who gets the Nobel prize? The whole credit system, as well as the notion of discovery, is called into question.

Linking the human genome sequencing project to the Watson and Crick story, as is routinely done, means also projecting a heroic discovery story on the more tedious work of sequencing. People have argued that this could help make the sequencing project look more exciting.

**JG:** What about the use of interviews in your research?

**SDC:** Interviews are extremely useful, but as historical sources you can't take them at face value. I have actually written about that—how to use interviews for writing the history of science, if you are interested!

Interviews happen usually in a dialog, and the account is really a co-production of these two individuals. A question is asked and a response given. So the response is directed in a certain way and it's always a reflection on past events, not a simple description of what happened in the past.

And then there is the way memory works. People get things completely wrong retrospectively. Sometimes there are conscious alterations, like cutting out details the interviewee prefers not to talk about. But memory also works selectively. And we forget things. A story, told many times, can become more real than the real thing. That is why a letter written at the time is generally more reliable a source than a memory of 30 years ago.

**JG:** But scientists keep a laboratory record of some kind.

**SDC:** Sometimes they are actually very surprised by what they find there! Because they have become convinced of the development of a particular series of events, but when they trace it back, they find it might not have happened the way they remember it.

Interviews for research purposes are very time-intensive. You have to work a lot in advance to be able to ask questions that will move beyond what people have written before. And then, after the interview, it's extremely time-consuming to transcribe it and then to check all the information. So I have done some but not too many interviews. I mainly view interviews as leads to historical research, to get to papers, to check interpretations. It's not the end product for me. It is just one part of the mosaic, one source among many others.

**JG:** What about the archives?

**SDC:** There are rules that govern the access to archives. In Britain, for instance, there is the 30-year rule for all government papers. Personal files are closed for 50 years beyond the death of the person. That's why there is always a lag time before historians take up a subject. And that's why many people think you can't do the history of the 1980s yet.

Kendrew featured quite strongly in my book because he deposited his very substantive collection of papers in the Bodleian [library at Oxford University] when he was still alive, and he collaborated in the production of the catalogue, which alone has about 900 pages! I needed his personal permission to get to the papers, and at the beginning he was rather restrictive. But with time I gained his trust, and by the end I had free access to practically everything.

These are the obvious things—personal papers and institutional papers—but then you have to look around. For instance, Max Perutz got a grant from the Rockefeller Foundation, so you go to their archives and see what's there. They have rich archives, including the diaries of their officers who regularly visited different laboratories and provided long descriptions of what they saw at different times.

Or you start being interested in the models of proteins and DNA and in the television programs about them. There is a wonderful BBC archives with correspondence between the producers and the scientists. You can trace the initiative for a specific program, and how the project changed along the way. What did scientists think about their collaboration? Were they fearful of the image they projected? This is science in the public realm that is moving far beyond science in the published scientific papers.

**JG:** Who were you thinking of as your audience?

**SDC:** This is a big issue in the history of science. Everyone feels that writing only for those five other historians of science who work in a similar field is not enough. Also, history of science is becoming so much a topic for popular writing. People speak about the “Sobel effect,” after Dava Sobel, the author of *Longitude,* which had such a big impact.

**JG:** That's a great book. I actually went to Greenwich a few weeks ago to see the Harrison clocks!

**SDC:** Historians also want to reach broader audiences. But you have to make sure the scholarship is not lost. Some people feel you have to do both, writing academic books and articles and more popular books.

The scientists are an audience I think we have to be especially concerned with because it is a huge market. Also, if we want to make an impact in the way science is perceived, then if the scientists themselves are not interested in what we write, there is a serious problem.

**JG:** To devote ten years of your work life to others' work, it strikes me that you would have to be drawn in to the characters. Perhaps you became quite fond of them.

**SDC:** I kept a distance, by design.

**JG:** I don't think, then, that I could be a historian of science!

**SDC:** It doesn't mean that you can't be enthusiastic about it. I say this in the preface of my book. Every time I come to my office, I pass by the old Cavendish laboratory, and in the summer especially there is always a group of tourists. As I pass by, I wait a bit to hear “Watson and Crick,” and so on, and that has become part of this cultural heritage. It was special writing that story here.

**JG:** I confess, I think I'm particularly drawn to the double helix discovery because it occurred during my birth year.

**SDC:** And I was born on the very day that famous photo of Watson and Crick with the double helix was taken!

We smiled at each other. And like a co-conspirator, she offered to show me the room shared by Watson and Crick when they toyed with their models. We went through the Cavendish portal, crossed a small courtyard, and turned right to enter the Austin wing, where the Department of Materials Science and Metallurgy is now housed. We slid up a set of stairs, hit the buzzer, and were admitted to a long corridor. There, three doors down on the left, was room 103, where a solitary woman sat, her back facing us. We matched up the verticals bands on the south wall with those in the backdrop of the photograph and convinced ourselves that this was the spot. The attraction was so strong, I found it hard to pull myself away. 

## 

**Figure:**
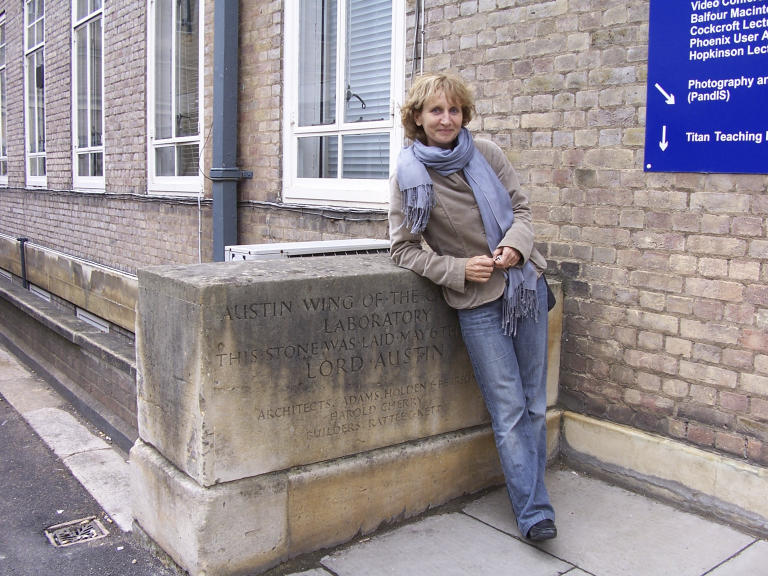
Soraya de Chadarevian

